# Flow Features of the Near Wake of the Australian Boobook Owl (*Ninox boobook*) During Flapping Flight Suggest an Aerodynamic Mechanism of Sound Suppression for Stealthy Flight

**DOI:** 10.1093/iob/obz001

**Published:** 2019-02-19

**Authors:** Jonathan Lawley, Hadar Ben-Gida, Krishnan Krishnamoorthy, Erin E Hackett, Gregory A Kopp, Gareth Morgan, Christopher G Guglielmo, Roi Gurka

**Affiliations:** 1 Department of Coastal and Marine Systems Science, Coastal Carolina University, Conway, SC 29579, USA; 2 Faculty of Aerospace Engineering, Technion, Haifa 32000, Israel; 3 Department of Civil and Environmental Engineering, University of Western Ontario, London,Ontario, Canada; 4 African Lion Safari, Cambridge, Ontario, Canada; 5 Department of Biology, University of Western Ontario, London, Ontario N6A 3K7, Canada

## Abstract

The mechanisms associated with the ability of owls to fly silently have been the subject of scientific interest for many decades and may be relevant to bio-inspired design to reduce noise of flapping and non-flapping flying devices. Here, we characterize the near wake dynamics and the associated flow structures produced during flight of the Australian boobook owl (*Ninox boobook*). Three individual owls were flown at 8 ms^−1^ in a climatic avian wind tunnel. The velocity field in the wake was sampled at 500 Hz using long-duration high-speed particle image velocimetry (PIV) while the wing kinematics were imaged simultaneously using high speed video. The time series of velocity maps that were acquired over several consecutive wingbeat cycles enabled us to characterize the wake patterns and to associate them with the phases of the wingbeat cycle. We found that the owl wake was dramatically different from other birds measured under the same flow conditions (i.e., western sandpiper, *Calidris mauri* and European starling, *Sturnus vulgaris*). The near wake of the owl did not exhibit any apparent shedding of organized vortices. Instead, a more chaotic wake pattern was observed, in which the characteristic scales of vorticity (associated with turbulence) are substantially smaller in comparison to other birds. Estimating the pressure field developed in the wake shows that owls reduce the pressure Hessian (i.e., the pressure distribution) to approximately zero. We hypothesize that owls manipulate the near wake to suppress the aeroacoustic signal by controlling the size of vortices generated in the wake, which are associated with noise reduction through suppression of the pressure field. Understanding how specialized feather structures, wing morphology, or flight kinematics of owls contribute to this effect remains a challenge for additional study.

## Synopsis

היכולת של ינשופים לעוף בצורה שקטה עוררה עניין מדעי רב במשך עשרות שנים ומהווה כיום מקור להשראה בתכנון כלי טיס מאוישים ובלתי מאוישים. בעבודה זו, אנו מאפיינים את שדה הזרימה בעקבה של הינשוף תוך כדי תעופה בנפנוף מתמיד. שדה הזרימה בעקבה (שובל) של שלושה ינשופים מסוג ינשוף נצי (*Ninox boobook*) נמדד במנהרת רוח סגורה מבוקרת לחץ וטמפרטורה, כאשר מהירות הרוח נקבעה על 8 מטר/שנייה. שדה הזרימה שנוצר בעקבה שהתפתחה מאחורי כנף הינשוף נמדד באמצעות מערכת מדידה מסוג High-speed Particle Image Velocimetry כאשר השדה נדגם בתדירות של 500 הרץ. מצלמת וידאו מהירה צילמה את הינשוף תוך כדי טיסה על מנת לאפיין את תנועת הכנף ואיפיון של מחזור הנפנוף. מאפייני ומבנה השובל אשר התפתחו בעקבה התקבלו ממדידת שדה המהירויות בזמן ובמרחב לאורך מספר מחזורי נפנוף רציפים. מדידת תנועת הכנף בו זמנית עם מדידת המהירות בעקבה אפשרה התאמה בין מאפייני השובל ושלבים שונים במחזור הנפנוף. נמצא כי שדה הזרימה בעקבה של כנף הינשוף שונה באופן משמעותי בהשוואה לזרימה בעקבות של ציפורים אחרות אשר נבחנו ונמדדו בתנאי ניסוי זהים באותה מנהרת רוח: חופית (*Calidris mauri*) וזרזיר מצוי (*Sturnus vulgaris*). השלת מערבולות המאפיינת שובלים של גופים בשדה זרימה לא נצפתה בעקבת הינשוף, במקומן נמדד שובל כאוטי ללא השלה מסודרת של מערבולות. השובל התאפיין בערכי ערבוליות נמוכות ובגדלי אורך ערבוב (אשר מאפיינים טורבולנציה) קטנים בהשוואה לשובל שהתפתח מאחורי הציפורים האחרות. מדידת המהירות אפשרה את חישוב שדה הלחצים בעקבה דרך שימוש במטריצת הסיאן (pressure Hessian) של הלחץ אשר מתקבל ממשוואות השימור של שדה הזרימה. שדה הלחצים בקירוב היה שווה לאפס בעקבה של הינשוף. אנו משערים כי הינשוף, תוך כדי טיסה, מתמרן את שדה הזרימה בעקבה אשר מתפתחת מאחורי כנפיו על מנת להנחית את הרעש האווירודינמי על ידי שליטה ובקרה של הערבוליות. שינוי הערבוליות בשדה גורם לשינוי בשדה הלחץ ואי לכך גורר באופן ישיר לשינוי ברמת הרעש האווירודינמי. מחקר נוסף נדרש על מנת לאפיין את תרומת המרכיבים הספציפיים כגון: מבנה הנוצות, מבנה הכנף, תנועת הכנף בנפנוף וכו׳, בשינוי אופיין הזרימה והנחתת הרעש בעקבה.

Características do fluxo de esteira proximal da coruja Ninox boobook durante o vôo batido sugerem mecanismo aerodinâmico de supressão sonora para vôo furtivo Características do fluxo de esteira proximal da coruja Ninox boobook durante o vôo batido sugerem mecanismo aerodinâmico de supressão sonora para vôo furtivo (Flow features of the near wake of the southern boobook owl (Ninox boobook) during flapping flight suggest an aerodynamic mechanism of sound suppression for stealthy flight) translated to Portuguese byG Sobralgabisobral@gmail.com Os mecanismos associados à capacidade das corujas de voar silenciosamente têm sido objeto de interesse científico há muitas décadas e podem ser relevantes para o biodesign ao reduzir o ruído de dispositivos voadores com flapping e não-flapping. Aqui, caracterizamos a dinâmica da esteira proximal e as estruturas associadas de fluxo produzidas durante o vôo da coruja Ninox boobook. Três corujas voaram em um túnel de vento climático a 8 ms - 1. O campo de velocidade na esteira foi amostrado a 500 Hz usando velocimetria de partícula por imagem (PIV) de alta velocidade e longa duração, enquanto a cinemática da asa foi visualizada simultaneamente usando vídeo de alta velocidade. A série temporal de mapas de velocidade que foram adquiridos ao longo de vários ciclos de batida de asas consecutivos nos permite caracterizar os padrões de esteira e associá-los às fases do ciclo. Descobrimos que a esteira produzida pela coruja era dramaticamente diferente de outras aves medidas sob as mesmas condições de fluxo (i. e., maçarico ocidental, Calidris mauri e estorninho comum, Strunus vulgaris). A esteira proximal da coruja não exibia qualquer desprendimento aparente de vórtices organizados. Em vez disso, foi observado um padrão de esteira mais caótico, no qual as escalas de vorticidade características (associadas à turbulência) são substancialmente menores em comparação com outras aves. Estimar o campo de pressão desenvolvido na esteira mostra que as corujas reduzem o Hessiano da pressão (i. e., a distribuição de pressão) para aproximadamente zero. Nossa hipótese é de que as corujas manipulam a esteira proximal para suprimir o sinal aeroacústico, controlando o tamanho dos vórtices gerados na esteira, que estão associados à redução de ruído através da supressão do campo de pressão. Entender como as estruturas especializadas das penas, a morfologia das asas ou a cinemática do vôo de corujas contribuem para esse efeito continua sendo um desafio para serem explorados em estudos adicionais.

সারাংশপেঁচার নিঃশব্দ উড্ডয়ন ক্ষমতার সাথে সম্পর্কযুক্ত পদ্ধতিগুলি বিগত কয়েক দশক ধরে বৈজ্ঞানিক আগ্রহের বিষয় হয়ে উঠেছে এবং যেটা ডানা ঝাপ্টানো এবং ডানা না ঝাপ্টাতে পারা উড্ডয়ন সক্ষম বস্তুগুলোর নিঃশব্দ উড্ডয়নে জৈব-অনুপ্রাণিত নকশা প্রণয়নে সহায়ক ভূমিকা পালন করতে পারে। এখানে আমরা উড্ডয়নরত দক্ষিনস্ত বুবুক পেঁচার (*Ninox boobook*) নিকটস্থ বায়ুঘুর্নীর গতি প্রকৃতি এবং বায়ু প্রবাহের কাঠামো বিশ্লেষন করেছি। তিনটি পৃথক পেঁচাকে জলবায়ু নিয়ন্ত্রনসক্ষম উইন্ড টানেলে ৮ মি/সে গতিতে উড়ানো হয়েছিল। দীর্ঘস্থায়ী উচ্চ-গতির কণা চিত্র ভেলোসিমেট্রি (PIV) ব্যবহার করে ৫০০ হার্টজ কম্পাংকে গতিবেগ ক্ষেত্র পরিমাপের নমুনাটি সংগ্রহ করা হয়েছিল এবং উইং কাইনেমেটিক্স উচ্চ গতির ভিডিও ব্যবহার করে একই সাথে চিত্রিত করা হয়েছিল। বিভিন্ন ধারাবাহিক উইংবিট চক্রগুলির ক্রমবর্ধমান সময়ের সাথে অর্জিত গতিচিত্র আমাদেরকে উড্ডয়ন পরবর্তী বায়ুঘুর্ণীর আকার, আকৃতি এবং বিন্যাস চিহ্নিত করতে এবং উইংবিট চক্রের পর্যায়গুলির সাথে সম্পর্কযুক্ত করতে সহায়তা করে। আমরা পর্যবেক্ষন করেছি যে পেঁচার উড্ডয়ন পরবর্তী বায়ুঘুর্ণীর আকার, আকৃতি এবং বিন্যাস একই গতিতে পরিচালিত অন্যান্য পাখির (যেমন ওয়েস্টার্ন স্যান্ডপাইপার *Calidris mauri*, এবং ইউরোপীয় স্টারলিং *Strunus vulgaris*) পরিক্ষনে প্রাপ্ত তথ্য-উপাত্ত এর চেয়ে উল্লেখযোগ্যভাবে আলাদা। পেঁচার ক্ষেত্রে উড্ডয়ন পরবর্তী নিকটস্থ বায়ুঘুর্ণীগুলো কোনো সুসংগঠিত ভরটেক্স শেডিং প্রদ্রর্শন করেনি এবং এর পরিবর্তে, আরো বিশৃঙ্খল বায়ুঘুর্ণীবিন্যাস পরিলক্ষিত হয়েছিল যেটায় ভর্টিসিটির আকৃতি (টার্বুলেন্স সম্পর্কিত) উল্লেখযোগ্যভাবে অন্যান্য পাখিগুলোর চেয়ে ছোট ছিল। উড্ডয়ন পরবর্তী বায়ুঘুর্ণীতে উতপন্ন চাপক্ষেত্র (যেমনঃ চাপবিন্যাস) পরিমাপ করে দেখা গিয়েছে যে পেঁচা চাপ হেজিয়ান কমিয়ে শুন্যর একেবারে কাছাকাছি নিয়ে আসে। আমরা ধারণা করি যে, পেঁচা উড্ডয়ন পরবর্তী বায়ুঘুর্ণী অঞ্চলে ভর্টিসের আকার নিয়ন্ত্রণ করে অ্যারোএকোস্টিক সংকেতকে প্রশমন করে যেটা চাপ ক্ষেত্র প্রশমনের মাধ্যমে শব্দ হ্রাসের সাথে সম্পর্কযুক্ত। পেঁচার বিশেষ পালক কাঠামো, পাখার অঙ্গসংস্থানবিদ্যা, অথবা ফ্লাইট কাইনেমেটিক্স এই নিঃশব্দ উড্ডয়নে কতটুকু ভূমিকা রাখে তা বুঝতে পারাটা ভবিষ্যত গবেষণার জন্য একটি চ্যালেঞ্জ।

布克鹰鸮在飞行过程中的尾流特征表明了无声飞行的空气动力学机制。摘要近十几年来, 猫头鹰无声飞行的机制一直都是科学研究的主题, 这启发了关于减少振动和无振动飞行装置噪音的设计。这篇文章中, 我们描述了在布克鹰鸮飞行过程中的尾流动力特征以及相关的气流结构。在风洞中, 三只猫头鹰以8 ms-1 速度飞行。当使用高速视频来记录翅膀动能时, 长时间高速粒子图像测速 （PIV） 也以500Hz对尾流的速度场进行采样。通过几个连续的翼拍周期, 我们获得了速度的时间序列场, 这使我们能够刻画尾流模式并且将它们与翼拍周期的相位相关联。在相同的气流条件下, 我们发现猫头鹰的尾与其他鸟类 （例如, 西方滨鹬、欧洲椋鸟、紫翅椋鸟） 显著不同。猫头鹰尾部并没有呈现出规律的漩涡减少。相反, 我们观测到的更混乱的尾迹模式, 涡度的特征尺度 (与湍流相关)明显小于其它鸟类。估测的尾流气压场表明猫头鹰能够降低气压海森矩阵（例如, 气压场的分布）到0。我们认为猫头鹰能够抑制压力场来降低噪声, 进而通过改变尾流来控制尾流中产生的涡旋大小, 进而抑制气动声学信号。理解如何猫头鹰的特殊羽毛结构、翅膀形状或飞行动力学产生这种效果仍然是进一步研究的挑战。

Las características de flujo de la estela cercana del búho de boobook del sur (Ninox boobook) durante el vuelo del aleteo sugieren un mecanismo aerodinámico de supresión del sonido para el vuelo sigiloso (Flow features of the near wake of the Australian boobook owl (Ninox boobook) during flapping flight suggest an aerodynamic mechanism of sound suppression for stealthy flight): translated to Spanish byJ Herasherasj01@gmail.com Los mecanismos asociados con la capacidad de los búhos para volar en silencio han sido objeto de interés científico durante muchas décadas y pueden ser relevantes para el diseño bioinspirado para reducir el ruido de los dispositivos de vuelo de aleteo y de no aleteo. Aquí, caracterizamos la dinámica de estela cercana y las estructuras de flujo asociadas producidas durante el vuelo de la búho boobook Australiano (Ninox boobook). Tres búhos individuales volaron a 8 ms - 1 en un túnel de viento climático aviar. El campo de velocidad en la estela fue muestreado a 500 Hz usando una velocimetría de imagen de partículas de alta velocidad (PIV) de larga duración, mientras que la cinemática del ala se fotografió simultáneamente usando video de alta velocidad. Las series de tiempo de los mapas de velocidad que se adquirieron en varios ciclos consecutivos de aleteo nos permiten caracterizar los patrones de estela y asociarlos con las fases del ciclo de aleteo. Descubrimos que la estela del búho era dramáticamente diferente de otras aves medidas en las mismas condiciones de flujo (es decir, Correlimos de Alaska, Calidris mauri y el Estornino Pinto, Strunus vulgaris). La estela cercana de la búho no mostró ningún desprendimiento aparente de vórtices organizados. En lugar, se observó un patrón de estela más caótico, en el que las escalas características de vorticidad (asociadas con la turbulencia) son sustancialmente más pequeñas en comparación con otras aves. La estimación del campo de presión desarrollado en la estela muestra que los búhos reducen la presión Hessian (es decir, la distribución de la presión) a aproximadamente cero. Nuestra hipótesis es que los búhos manipulan la estela cercana para suprimir la señal aeroacústica controlando el tamaño de los vórtices generados en la estela, que están asociados con la reducción de ruido mediante la supresión del campo de presión. La comprensión de cómo las estructuras de plumas especializadas, la morfología de las alas o la cinemática de vuelo de los búhos contribuyen a este efecto sigue siendo un desafío para un estudio adicional.

**தென்னக பூபூக் ஆந்தையின் (Ninox boobook) இறக்கைப் பின்னியங்கும் காற்றுப் புலக் கூறுகளின் பண்புகள் பரிந்துரைக்கும், இரைச்சலற்ற-பதுங்குப் பறத்தலுக்கான காற்றியக்கவியல் இயந்திரநுட்பங்கள்**

ஆந்தைகளின் இரைச்சலற்றப் பறக்கும் திறனின் பின்னால் இருக்கும் அறிவியல்/இயக்கவியல் ஒரு முக்கிய ஆராய்ச்சி ஆர்வமாக பல ஆண்டுகளாக விளங்குகிறது. மேலும், குறைவான இரைச்சலை உடைய நிலையான இறக்கை அல்லது சிறகடிக்கும் இறக்கைகளுடையப் பறக்கும் எந்திரங்களை வடிவமைக்க அறிவியல் உந்துதலைக் கொடுத்துவருகிறது. இந்த ஆராய்ச்சிக் கட்டுரையில், தென்னக பூபூக் ஆந்தை என அறியப்பட்ட Ninox boobook பறக்கும் பொழுது அலைக்கழிக்கப்படும் காற்றின் இயக்கங்கள் மற்றும் அதனோடு தொடர்புடைய காற்று வடிவக்கூறுகள் ஆகியவற்றின் பண்புகளைத் தொகுத்து விவரித்துள்ளோம். பறவைகளுக்காக வடிவமைக்கப்பட்ட, காலநிலை மாறுபாடுகளை பிரதிபலிக்கும் திறனுடைய காற்றுக்சுரங்கையில் மூன்று தனித்துவமான ஆந்தைகள் நொடிக்கு 8 மீட்டர் வேகத்தில் பறக்க விடப்பட்டன. நெடுநேர, அதிவேக particle image velocimetry (PIV) என அறியப்பட்டத் தொழில்நுட்பத்தைப் பயன்படுத்தி நொடிக்கு 500 படங்கள் வீதம் காற்றின் திசைவேகப்புலங்கள் பெறப்பட்டன. அதனோடு இணையான இயங்கியலை அறிவதற்காக, ஆந்தைகளின் சிறகடிக்கும் இறக்கைகள் தொடர்ச்சியாக அதே அதிவேக வீதத்தில் படம்பிடிக்கப்பட்டன. இத்தகைய பல இறக்கைச்சுழற்சிகளுக்கு பெறப்பட்ட காலத்தொடர் திசைவேக விவரணைகள், காற்று இயக்க வடிவங்கள் மற்றும் கூறுகளை வகைப்படுத்தி, அதை உருவாக்கிய குறிப்பிட்ட நேரத்தில் இறக்கையின் அமைவு மற்றும் நிலையுடன் தொடர்பு படுத்தி பண்புகளை விவரிக்க நமக்கு வாய்ப்புகளை வழங்குகிறது. இதே காலநிலை மற்றும் வேக நிலைத் தேர்வுகளின் படி ஆராயப்பட்ட மற்றப் பறவைகளிருந்து (மேற்கத்திய உள்ளான், Calidris mauri மற்றும் ஐரோப்பிய நாகணவாய், Strunus vulgaris), ஆந்தைகளால் அலைக்கழிக்கப்பட்ட காற்றின் பண்புகள் வியத்தகு அளவிற்கு மாறுபட்டிருப்பது இந்த ஆராய்ச்சியில் கண்டுபிடிக்கப்பட்டது. ஆந்தையின் இறக்கையின் அருகில் உள்ள காற்றுப்புலம், அடுக்கப்பட்ட தொடர்ச்சியான சுழலும் காற்று இயக்க கூறுகளை வெளிப்படுத்தவில்லை. மாறாக காற்றுப்புலமானது மிக ஒழுங்கற்ற சிக்கலான சுழல் காற்றுக் கூறுகளைக் கொண்டிருந்தது. மேலும், இந்த சுழல்காற்றுக் கூறுகளின் பான்மை அளவுகள், மற்றப்பறவைகளினுடன் ஒப்பிடும் பொழுது கணிசமாகக் குறைவாக உள்ளது. தொடர்ந்து, காற்றழுத்தப் புலங்களை அளவீடு செய்து பார்க்கும்பொழுது, ஆந்தைகள் காற்றழுத்த (pressure Hessian) பகிர்தலை/பரவலை ஏறத்தாழ சுழியமாகக் குறைக்கின்றன எனப் புலப்படுகிறது. இங்கனம் நாங்கள், ஆந்தைகளானவை அவற்றின் பின்னியங்கும் காற்றுப்புலத்தைத் திறமையாகக் கையாண்டு சுழல்காற்றுக்கூறுகளின் பான்மை அளவுகளைக் கட்டுப்படுத்துவதின் மூலம் காற்றழுத்தப் புலத்தின் பகிர்தலைக் குறைத்து காற்றொலிசார் சமிக்கைகளை ஒடுக்குகின்றன என அனுமானிக்கிறோம். இத்தகைய காற்றொலிசார் சமிக்கைகளே இரைச்சலுடன் தொடர்புடையவை. தொடரும் ஆராய்ச்சிக்கு, ஆந்தைச் சிறகுகளின் சிறப்பான வடிவமைப்புகள், அவற்றின் இறக்கை வடிவமாற்றுப்பண்புகள் மற்றும் இறக்கை இயங்கியல் ஆகியவற்றைப் புரிந்துகொள்ளுதல் அடிப்படையாகவும் சவாலாகவும் அமைகிறது.

## Introduction

Owls are exceptional hunters due to the fact that most species of the order Strigiformes share the common characteristic of flight which is nearly inaudible to humans and, more importantly, to their prey (Mascha 1904; Dubois 1924; Neuhaus et al. 1973; Bachmann et al. 2007; Sarradj et al. 2011). Owl’s stealth mechanisms (Thorpe and Griffin 1962) function during both flapping and gliding flight modes. There are many potential contributors to this specialized feature of the owls such as wing morphology, unique feathers structures, and flight behavior (Graham 1934). Their typical gliding Reynolds numbers are within the intermediate range [O(10^5^)], therefore, low Reynolds number theories (Weis-Fogh 1973) cannot predict their unique flight pattern. In addition, owls feature highly maneuverable low-speed gliding flight capabilities (Johnsgard 1988), yet their aerodynamic performance (lift–drag ratio) has been suggested to be relatively low (Kroeger et al. 1972; Geyer et al. 2017).

The most extensive area of research has been focused on the role of the feathers (Graham, 1934); both on their material properties including flexibility as well as their geometrical ones such as location and spacing. The material property most studied is the velvety surface of the flight feathers. Graham (1934) observed that these velvety feathers (sometimes referred to as downy) feature long hairs and barbules, which he hypothesized would muffle any rustling noise associated with feathers rubbing together. He also suggested that these feathers act as a sound absorber, which would dampen out any small vibrations near the wing. Studies modeling the velvety feathers with an artificial surface that mimics the feathers (e.g., velvet) have shown that the separation bubble is reduced, delayed, or eliminated altogether (Klän et al. 2009, 2012; Winzen et al. 2013). Winzen et al. (2015a, 2015b) showed that the velvety feathers as well as the flexibility of the wing stabilize the flow field at low Reynolds numbers, enabling the owl to fly more slowly.

Another unusual aspect of the owl wing is the leading-edge serrations: a comb of evenly-spaced barbs along the wing leading-edge. Graham (1934) emphasized the importance of the leading-edge serrations as a major noise reducer. Commonly, the flow past the leading-edge over the wing surface may be separated depending on the wing’s angle of attack and its camber. These serrations have been found to stabilize the flow (Winzen et al. 2014) and promote the development of a leading-edge vortex (LEV) (Lowson and Riley 1995) that can augment lift (Geyer et al. 2017). Kroeger et al. (1972) and Anderson (1973) suggested a mechanism to achieve this that involved redirecting the flow passing through the serrations toward the wingtip, which they proposed delays flow separation and produces non-linear lift on the outer half of the wing. Rao et al. (2017) demonstrated that leading-edge serrations play a crucial role in sound suppression at the expense of low aerodynamic performance at low angles of attack.

The fringes along the trailing-edge of the wing have also been studied in connection with the silent flight of owls. Once the flow passes the wing, it is shed from the trailing-edge toward the wake region. Former studies show that these fringes can manipulate the aerodynamic noise (Bachmann et al. 2012; Geyer et al. 2012; Jaworski and Peake 2013). Geyer et al. (2012) conducted acoustic wind tunnel experiments on prepared wings from a variety of bird species and concluded that the silent flight of owls is a consequence of their special wing and feather adaptations.

Relative to the role of feathers (Wagner et al. 2017; Weger and Wagner 2017), investigations of the wake characteristics, and their relation to quantities like pressure that influence noise generation, are sparse. In particular, wake measurements of freely flying owls that account for all of the aforementioned feather features, along with flying behavior, are lacking. The wake signature provides information on flight performance (i.e., aerodynamic forces) and sheds light on the wing–wake interaction and associated flow mechanisms (Chin and Lentink 2016).

The distribution of the pressure field in the wake results from its flow dynamics. The differential form of the momentum equations (Navier–Stokes) describes the coupling between the pressure and the velocity fields. The formation of the wake flow region stems from the two shear layers moving across the airfoils and interacting beyond it. The velocity field in the near wake region is governed by a streamwise velocity deficit that is formed from the merger of the two-shear layers. The velocity deficit indicates high level of shear in the wake region, as well as vorticity. When the Reynolds number is relatively high, the wake becomes turbulent and vorticity plays a major role in the flow dynamics.

Since the pressure is coupled with velocity, it is also coupled with velocity gradients. It is sometimes convenient to express the pressure as a function of the velocity field by taking the divergence of the Navier–Stokes equation and applying the continuity equation. This results in an expression that is similar to the Poisson equation (Corcos 1964). Re-arranging the equation can yield an expression for the pressure as a function of vorticity and strain, known as the pressure Hessian. The noise generated in the wake, which is commonly referred to as aerodynamic noise (or aeroacoustics), is a function of the velocity field and its nature (Lighthill 1952, 1954). The sound waves are essentially pressure waves and the aerodynamic noise is governed by the pressure distribution within the flow. Pressure and density perturbations generate pressure waves which are correlated with the sound waves. Since pressure and velocity are coupled, in order to characterize the aeroacoustics signature of the owl, one needs to study the wake dynamics.

Advances in technology have allowed for quantitative measurements of wakes using particle image velocimetry (PIV). This method has been applied to a number of flying species (i.e., Spedding et al. 2003; Hedenström et al. 2006; Rosén et al. 2007; Henningsson et al. 2008; Johansson and Hedenström 2009; Tobalske et al. 2009; Altshuler et al. 2009; Hubel et al. 2010; Muijres et al. 2012; Kirchhefer et al. 2013; Gurka et al. 2017), and measurements have been made in both the far wake (Spedding et al. 2003; Hedenström et al. 2006; Rosén et al. 2007; Henningsson et al. 2008; Johansson and Hedenström 2009; Tobalske et al. 2009; Altshuler et al. 2009; Hubel et al. 2010; Muijres et al. 2012) and near wake (Kirchhefer et al. 2013; Gurka et al. 2017). All former wake studies of birds have demonstrated organized shedding in the wake during flapping flight (see details of their data collection and the specimens tested in Supplementary 1). The organized pattern in the wake of flapping birds wing is dominated by the shear layers generated above and below the wing, coupled with root and tip vortices that merge into the wake region, branching a set of vortex lines that dominate the wake. The organized shedding enables estimation of aerodynamic forces, such as lift where Kutta–Joukowski theorem can be applied to estimate it during flight (Henningsson and Hedenström 2011). These unique measurements in the wake of freely flying birds are both difficult to obtain and imperative to the advancement of our understanding of the different specialized characteristics of various bird species. They provide critical insight needed to compare to theoretical models and visualizations of the wake dynamics of flying birds (i.e., Rayner 1979a, 1979b). It is noteworthy to mention that these conceptual models (Rayner and Gordon 1998) assume that the wake flow exhibits some sort of organized motion and are idealized.

Recently, Doster et al. (2014) flew a trained barn owl (*Tyto alba*) in a long hallway, where they performed stereo-PIV measurements around the owl during flight. They showed, qualitatively, that a complex vortex flow system was developed in the wake during flapping flight. However, a well-defined topographical characterization of the wake of an owl and a quantitative estimation of the aerodynamic forces exerted on it are lacking, either experimentally or numerically.

In this study, PIV measurements in the near wake of a freely flying boobook owl (*Ninox boobook*) were performed and the results presented herein provide unique insight into the aerodynamics of owls and the interaction between their unique wing morphology and wake flow dynamics. We compare the wake characterization to two other species that we have measured under the similar conditions: a passerine songbird: European starling (*Sturnus vulgaris*), and a non-passerine shorebird: western sandpiper (*Calidris mauri*). We use wing kinematics from video and wake reconstructions from PIV measurements to compute characteristic spatial flow scales of the wakes. We show that the characteristic spatial scales of the wake relative to chord size of the owl are smaller than those of the other two species and that the owl exhibits the smallest pressure Hessian. Because the near wake flow affects the pressure field within the wake, and the pressure field is related to the aeroacoustic noise (Lighthill 1952), one can assume that the wake characteristics play a major role in the aeroacoustic variations.

## Materials and methods

### Birds

Two male and one female captive reared boobook owls (*N.boobook*) were obtained from the raptor program of the African Lion Safari in Cambridge, Ontario, Canada and brought to the Advanced Facility for Avian Research (AFAR) at the University of Western Ontario, London, Ontario, Canada under animal protocols from the University of Western Ontario Animal Care Committee (UWO #2010-216) and the African Lion Safari (BOP-15-CS). Morphological parameters of the owls, as well as the non-dimensional flow numbers associated with the performed experiments are summarized in Table 1.

**Table 1:** Morphological characteristics of the birds flown along with the characteristic flow numbers for the experiments performed at AFAR

Owl	Mass (g)	Wing span (cm)	Root chord (cm)	Semi-span (cm)	Body length (cm)	Body width (cm)	Wingbeat frequency (Hz)	Reynolds number	Strouhal number	Reduced frequency
1	273	90	13	40	30	10	6	66,200	0.35	0.31
2	300	78	14	34	31	10	6	71,300	0.37	0.33
3	255	70	14	30	31	9	6	71,300	0.37	0.33

Over several days the birds were brought to the AFAR in the morning and flown the same day after a brief acclimation. The owls were handled by a professional trainer (G.M.) but were not systematically trained in advance of experiments. Rather they flew steadily for several seconds on each attempt, long enough to capture image sequences suitable for PIV and kinematic analysis. Although the flights took place during daytime the wind tunnel was dark so that PIV could be performed. Optoisolators operated by six infrared transceivers were integrated into the PIV system (upstream from the laser sheet location) in order to prevent direct contact between the bird and the laser sheet (Kirchhefer et al. 2013). The optoisolators triggered the laser only when the owl was flying upstream from the PIV field of view (FOV). The isolation from the laser sheet and triggering system ensured the safety of the birds.

### Wind tunnel

The owls were flown in the hypobaric climatic wind tunnel at the AFAR [see Kirchhefer et al. (2013) for more details]. The wind tunnel is a closed loop type with a glass octagonal test section of 2 m length, 1.5 m width, and 1 m height preceded by a 2.5:1 contraction. The turbulence intensity at the test section was smaller than 0.3% with a velocity profile uniformity of 0.5%. Speed, pressure, temperature, and humidity can be controlled to generate various flight conditions at different altitudes. The birds were introduced into the test section through a 0.5 m open jet section located between the downstream end of the test section and the diffuser. The flight conditions for all the owls were at ground level: atmospheric pressure, a temperature of 15°C, and a relative humidity of 80%. The wind tunnel speed during the owls’ flight was set to 8 m/s. Using a projected cross-section of the owl (#1 in Table 1), conservative estimate of the blockage ratio is below 3.5%. We assume that wall effects due to interaction between the wing tip and the wind tunnel wall were negligible given that the minimum gap between the wall and the wing tip was about 40 cm, which is much larger than the flow scales characterized by the wing chord (∼13 cm).

### Particle image velocimetry

**Fig. 1:**
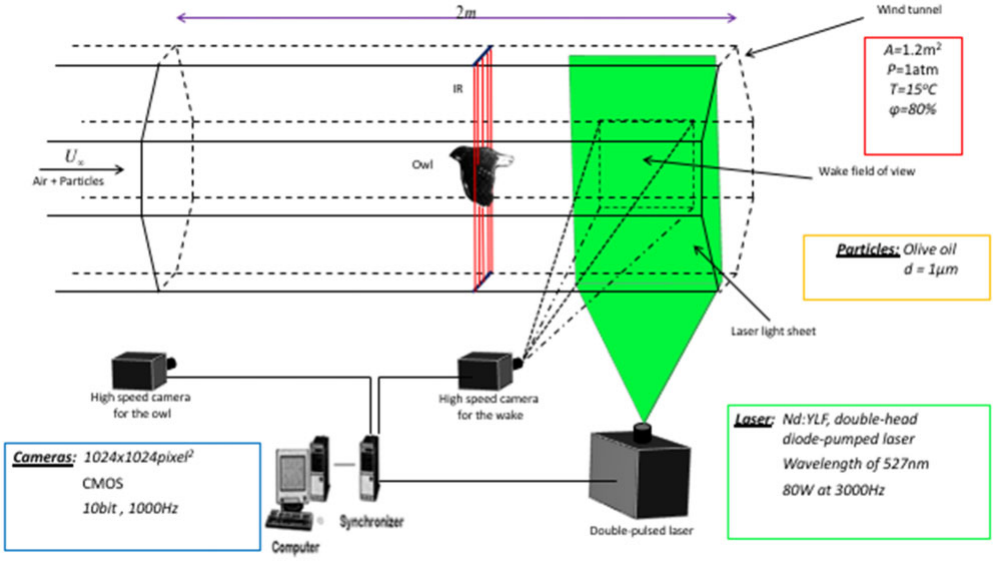
Illustrative scheme of the hypobaric wind tunnel and the experimental setup systems comprised of long-duration time-resolve PIV and high-speed imaging.

A long-duration time-resolved PIV system (Taylor et al. 2010) was employed to measure the near wake field behind the owls’ wings during flight. A schematic description of the PIV setup and the hypobaric wind tunnel at AFAR are shown in Fig. 1. The PIV system consisted of a 80 W double-head diode-pumped Q-switched Nd:YLF laser operating at a wavelength of 527 nm and two CMOS cameras (Photron FASTCAM-1024PCI) with spatial resolution of 1024× 1024 pixel^2^ operating at a rate of 1000 Hz and a 10 bit dynamic range. The PIV system was capable of continuously acquiring image pairs at 500 Hz using two cameras for up to 20 min. Olive oil particles, 1 μm in size were introduced into the wind tunnel using two Laskin nozzles (Echols and Young 1963) at the downstream end of the test section; thus, it did not cause a disturbance to the flow or to the bird.

**Fig. 2:**
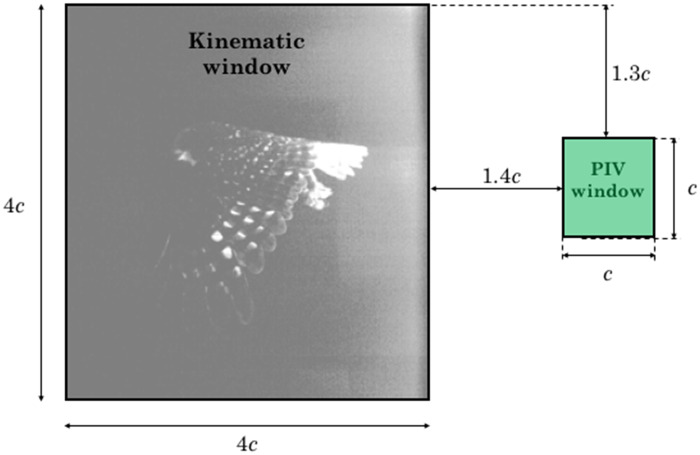
PIV and kinematic imaging fields of view (FOV). The locations of the measured FOVs are at the center of the tunnel, observing a streamwise-normal plane. The PIV FOV was 13×13 cm^2^ and the kinematic FOV was 52×52 cm^2^. The distance between the two FOVs was 18–19 cm.

One of the CMOS cameras was used for the PIV, while the other CMOS camera was used for measuring the wingbeat kinematics simultaneously with the PIV. The PIV camera’s FOV was 13× 13 cm^2^ corresponding to 1*c*×1*c*, where *c* is the owl mean chord length (an average of the three owls’ chord lengths) and the kinematic camera’s FOV was 52× 52 cm^2^ corresponding to 4*c*×4*c*, as shown in Fig. 2. The near wake flow field was sampled in the streamwise-normal plane with temporal resolution of 500 Hz and the distance from the owl’s trailing-edge to the FOV varied between 1.4 and 4 chord lengths. Multiple experiments were conducted to sample the near wake flow field at different locations along the wing span of the owl, which enabled us to comparatively analyze the wake characteristics developed at different wing sections along the span at each wingbeat. The velocity fields were computed using OpenPIV (Taylor et al. 2010) with 32× 32 pixel^2^ interrogation windows and 50% overlap, yielding a spatial resolution of 64 vectors per average chord, equal to 1.8 vectors per mm. The average pixel displacement was about 6 where the time between two exposures was set to 90 μs. The coordinate system used is a right-handed Cartesian system, where *x*, *y*, and *z* corresponds to the streamwise, normal, and spanwise directions. *x* is directed downstream, *y* is directed upward, and *z* is determined according to the right-hand rule. The streamwise and normal velocity components are denoted by *u* and *v*, respectively.

### Estimation of owl wing kinematics’

Wingbeat kinematics were recorded using one of the high-speed CMOS cameras as described above. From the acquired images, we calculated the wingbeat frequency, wingbeat amplitude, and angle of attack. The kinematic images were synchronized with the PIV images in order to provide a direct relationship between the wake formed by the wing motion and its kinematics.

The wake locations with respect to the trailing-edge of the wing during flight were determined from both captured images and the distance between them as depicted in Fig. 2. To determine the location of the light sheet along the bird’s wing or body, a 30 Hz CCD camera with 1600× 1200 pixel^2^ resolution was mounted downstream of the test section pointing toward the location where the bird would trigger the laser and taking spanwise-normal plane images. A spatial calibration was performed before the experiment. Once synchronized, spanwise positions were assigned to the wake data acquired at 500 Hz based on interpolation from the simultaneously recorded spanwise positions. These images enabled us to identify the location of the light sheet relative to the wing during the experiments and determine its location with respect to the body center. This information allowed us to couple the wake flow features with the wing morphology: wingtip, primary and secondary remiges, and close to the body (at the root).

We extracted the wingtip motion using motion analysis software, Kinovea (https://www.kinovea.org). A point close to the wingtip was tracked for all owls over the continuous wingbeat cycles. For each data set, the tracking identification for the kinematics analysis was located at the tip of the primary feather eight (P8, the third wing feather from the distal end of the wing). The number of wingbeat cycles was calculated by normalizing the total evaluation time with the wingbeat period.

### Flow experiments

Table 2 summarizes the collected PIV data sets obtained during the experiments for the three owls that were analyzed in this study. Only successful owl flights were recorded. A successful flight refers to an experiment where the owl triggered the laser and the PIV system acquired images of the flow field simultaneously with the wing kinematics. The data presented herein correspond to sets (“scenes”) where the owl did not accelerate or decelerate and maintained altitude during flapping mode. A total of nine scenes are presented, where each scene consists of hundreds of vector maps that correspond to multiple consecutive wingbeat cycles during free flight. This large set of data enabled us to statistically characterize the near wake flow field and its interaction with the owl’s wing. The PIV measurements were taken behind the owl’s wing where a wake was present, and the wing motion was clearly identified in the kinematic images.

**Table 2:** A summary of the datasets collected during the owl flight experiments

Owl	Experiment number	Scene number	Number of vector maps	Total number of identified^a^ wakes	PIV downstream^b^ location (*c*)	Light sheet location^c^
2	1	135	318	150	3.8	Between the primary and secondary remiges
2	2	136	304	100	2.1	Between the primary and secondary remiges
3	3	137	480	125	2.4	Center of the primary remiges
3	4	141	271	100	1.6	Between the primary and secondary remiges
1	5	146	410	100	3.3	At the tip of the primary remiges
3	6	150	150	120	2.9	Close to the root at the secondary remiges
1	7	153	770	100	4.2	At the tip of the primary remiges
1	8	158	912	195	3.9	At the tip of the primary remiges
1	9	159	813	250	2.1	Between the primary and secondary remiges

a Indicate the number of maps where both wing kinematics and flow field were successfully analyzed out of the total number of vector maps per experiment.

b The distance from the trailing edge where the wake was captured.

c With respect to the wing spanwise location.

An error analysis based on the root sum of squares method was applied to the velocity data and the wing kinematics, following Gurka et al. (2017). The errors were estimated as: 2.5% for the instantaneous velocity values, 10% for the instantaneous vorticity, and 4% for the circulation, which was calculated, based on the vorticity field (Raffel et al. 2007). The error introduced in the kinematic analysis resulted from the spatial resolution of the image and the lens distortion leading to an estimated error of 5% in the wing displacements.

### Blob analysis

In order to characterize the differences within the wake flow patterns of the birds studied herein, a quantitative comparison based on patterns topography was performed. For the topographical analysis, we utilize the so-called “blob” analysis. The motivation of this analysis is to characterize the dominant spatial scales in the reconstructed wake. The vorticity contours are presented in Fig. 5a here, and in Figs. 4 and 5 in Gurka et al. (2017). All the contours plotted employ a threshold of −1 and +1 for the normalized vorticity. The blob analysis essentially calculates the area of the concentrated vorticity regions (*ω_z_c*/*U_∞_* <−1 and *ω_z_c*/*U_∞_* > 1) and computes a histogram of these areas. A more detailed description of the procedure can be found in the Supplementary Material, Supplementary 3. For brevity, the analysis transformed vorticity contour images to grayscale images and removed the background. The grayscale image was filtered and then converted into a binary image. The binary image was evaluated for interconnectivity of non-zero pixels using a connectivity of eight nodes. The sums of the connected pixels were used to compute an area along with the image calibration from the PIV measurements. The histogram of these areas was plotted and power density functions were fit to the histograms as further described in the wake topography characterization subsection.

### Flow scale analysis

In order to characterize the scales of the flow in the near wake region and their dependency on the flapping wing motion we choose to utilize a known statistical approach when estimating the integral lengthscale in case of turbulent flow (Pope 2000). In order to estimate the fluctuating part of the flow, we applied a local Galilean decomposition; i.e., *u′* = *u*–*u*_avg_, *v*′ = *v*–*v*_avg_ where *u*_avg_ and *u*_avg_ are the spatially averaged (over the PIV FOV) velocities of the velocity components examined over the direction of the correlation. A similar technique was applied to PIV data in shear flows to remove the convection velocity (Adrian et al. 2000). The analysis does not attempt to estimate turbulent properties based on this decomposition; but rather is used to calculate the auto-correlation values for a fluctuating portion of the velocity field, which presumably is associated with turbulence. The longitudinal scale corresponds to the result obtained from correlating the velocity component along the same direction and calculating the area under the normalized correlation curve. Correlating the velocity components along the normal direction yields the transverse scales.

### Pressure Hessian

The pressure Hessian is a key quantity that controls vortex stretching through interactions associated with the pressure term (Ohkitani and Kishiba 1995; Tsinober 2013) in the momentum equations for fluids. The pressure Hessian is computed by applying a divergence to the incompressible Navier–Stokes equations:

(1) $$\nabla^{2}p=\rho \left(\frac{1}{2}\omega^{2}-s^{2}\right),$$

where *p* is the pressure, *ω*^2^ =*ω_i_ω_i_*, *s*^2^ =*s_ij_s_ij_*, *ω_i_* is the vorticity vector, *s_ij_* is the strain rate tensor, and *ρ* is the fluid density. Note that because the data are comprised from two-dimensional flow field measurements, we can only estimate the corresponding terms contributing to the pressure Hessian. We account for the spanwise vorticity component and the rate of strains in the streamwise and normal directions. While this term provides an insight to the relation between vorticity and strain, it also provides an indirect estimate of the pressure field developed within the flow.

## Results

### Owl wing kinematics

**Fig. 3:**
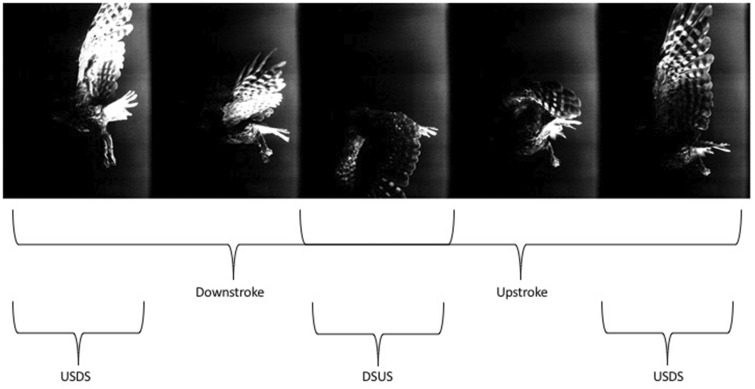
Sequence of instantaneous images showing a full wingbeat cycle of the boobook owl as the owl moves from right to left as it did in the wind tunnel.

**Fig. 4:**
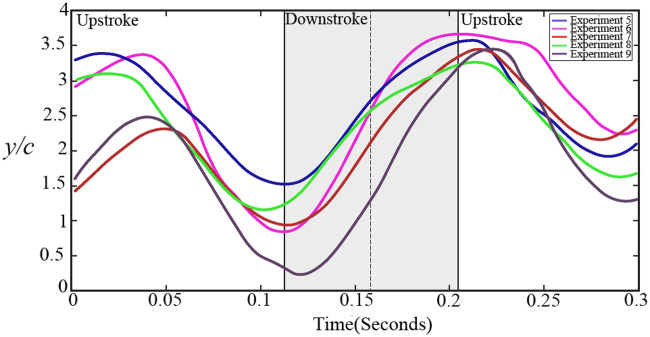
Wingbeat kinematics: non-dimensional amplitude of the wingtip versus time. The solid vertical lines illustrate the wings transition phases. The dashed line illustrates the points during the DS where the angle of attack was calculated.

We use a similar approach as Gurka et al. (2017), which followed guidelines suggested by Wies-Fogh (1973), where the wingbeat cycle was divided into four distinct phases: upstroke (US), transition from US to downstroke (USDS), DS, and transition from DS to US (DSUS). Following an analysis of the owl wing’s kinematics as described in the “Materials and methods” section, Fig. 3 presents images in sequential order from right to left as an owl flies through one full wingbeat cycle during forward flight (upstream, against the wind). We estimated that for the various flight durations, the average frequency was about 6 Hz (Fig. 4). Therefore, the corresponding Strouhal number for owls #2 and #3 was 0.37 and for owl #1 was 0.35. Herein, the Strouhal number is defined as *St* =*fA*/*U_∞_*, where *f* is the flapping frequency, *U_∞_* is the speed of flight (wind tunnel speed), and *A* is the peak-to-peak cross stream amplitude of the motion (Anderson et al. 1998). These values suggest that the owls’ wings, according to Anderson et al. (1998), may generate leading edge vortices during flapping flight. A semi-sinusoidal pattern is observed, covering the US, DS, and transition phases over almost two consecutive wingbeat cycles. The axes presented are normalized by the chord length. The trend is similar for various data sets covering flights of the three owls. The solid vertical lines define the transitions between the wingbeat phases whereas the dashed line illustrates the point during the DS phase where the angle of attack was calculated. It is assumed that during the flight, the owl wing operates at relatively high angles of attack, which is common in other birds as well (Shyy et al. 2008). Operating at high angles of attack at their characteristic Strouhal numbers allows owls to fly more slowly while still being able to generate lift (Rosti et al. 2017) through the formation of LEV (Anderson 1973).

### Near wake fluid dynamics

The near wake of the owl provides insight into the flow–owl interaction. The owl wakes differ qualitatively from those of other birds that have been measured (by us and others), which are more similar to each other. In order to qualitatively assess the wake evolution resulting from the owl in flapping flight, we followed the same procedure as originally suggested by Spedding et al. (2003) and utilized later for other passerines and shorebirds (Kirchhefer et al. 2013; Gurka et al. 2014). The wake evolution in time, which can be transformed into the evolution in space enables one to observe how the vortical patterns in the wake region provide a unique signature of a bird’s flight. The wake reconstruction procedure we used is described in detail in Gurka et al. (2014). Throughout the presented wingbeat cycles, the owls’ position did not change much relative to the measurement plane. Therefore, Taylor’s hypothesis (1938) is applied, following the assumption that the flow remains relatively unchanged as it passes through the measurement plane. The utilization of the long-duration time-resolved PIV system enabled the reconstruction of the wake evolving behind the wings. The owls flew from right to left (Fig. 3); therefore, the downstream distance is measured as positive chord lengths. What appears as downstream essentially happened earlier while what appears as upstream happened later. Each wingbeat cycle corresponds to five to eight cord lengths for the various wakes analyzed. Each individual scene analysis corresponds to 0.5–2 wingbeat cycles; thus, we can analyze the flow field behind the owl continuously and identify trends within the flow patterns.

**Fig. 5:**
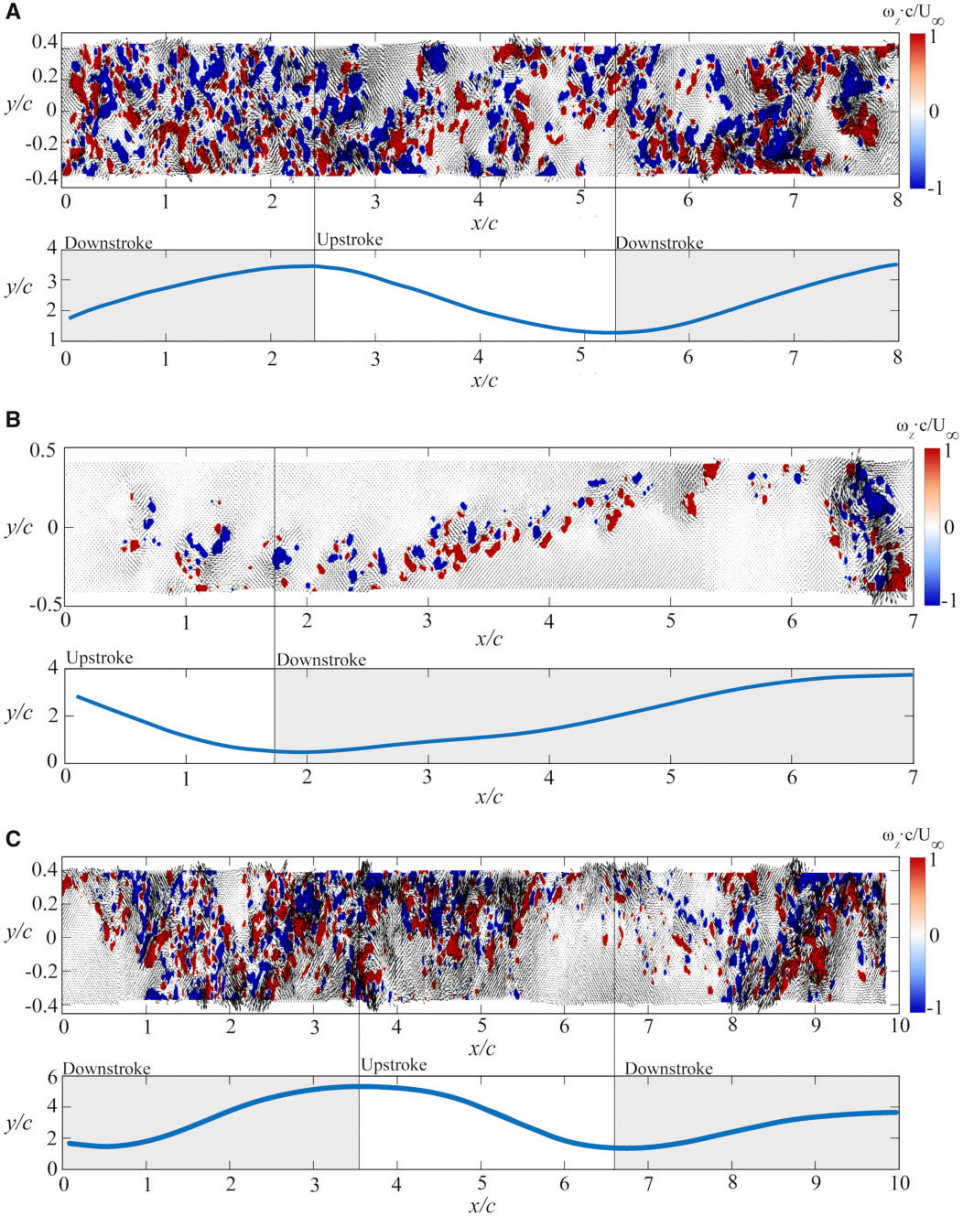
Near-wake flow features of the boobook owl while flying in a flapping mode. The owl flew from right to left. (i) Wake reconstruction—the wake was sampled behind the wing at different spanwise sections: **a**) between the primary and secondary remiges; Experiment #9, **b**) outer region of the wing; the furthest location in the primary remiges; Experiment #8 and **c**) Middle of the primary remiges; Experiment #3. Contours represent the values of spanwise vorticity and the velocity vectors superimposed on the contour maps depict the two-dimensional, two-component velocity field in the near wake. The regions of positive and negative vorticity are marked in red and blue, respectively. The spanwise vorticity was normalized by the ratio between the chord length *c*, measured at the semi-span location (between the primary and secondary remiges) and the free-stream velocity (i.e., the flight speed), *U_∞_*. (ii) Wingtip displacement—the wingtip displacement is plotted against downstream chord length to directly correlate with the respective wake. The vertical black lines in each graph represent the transition from US to DS or DS to US, respectively. All the wakes have been calculated based on the same threshold of the normalized vorticity values (−1 to +1).

The evolution of the near flow wake behind the freely flying owls is depicted in Fig. 5. It appears that the shedding of vortices from the wing are somewhat lacking coherence or consistency where one would expect to observe some sort of shedding behavior; organized or non-organized from a propulsive wake (as can be observed during birds’ flight, i.e., Spedding et al. 2003; Hedenström et al. 2006; Rosén et al. 2007; Henningsson et al. 2008; Johansson and Hedenström 2009; Tobalske et al. 2009; Altshuler et al. 2009; Hubel et al. 2010; Muijres et al. 2012; Kirchhefer et al. 2013; Gurka et al. 2017). The vorticity patterns in the wake appears disorganized, as shown in multiple sets of the data for the three owls investigated (Fig. 5). Figure 5a depicts the wake reconstruction from data taken in Experiment 9. The wake presented corresponds to the flow formed above and below the wing section, located between the primary and secondary remiges. Additional datasets acquired at the same location are shown in the Supplementary Material for Experiments 1, 2, 4, and 7 (see Supplementary 2). Experiment 8, presented in Fig. 5b, corresponds to the wake formed at the outer region of the wing; the furthest location in the primary remiges, where a tip vortex is present. The tip vortex appears as a concentrated spanwise vorticity region, almost circular in its geometrical shape marked with strong positive and negative vorticity values preceded by weak shedding that occurs over the entire wingbeat cycle. Additional datasets acquired at the same location are shown in the Supplementary Material for Experiments 5 and 7 (see Supplementary 2). It is noteworthy that Experiment 7 had mixed wake flow patterns, which may indicate that the owl was moving in the spanwise direction during flight. Figure 5c depicts the wake behind the middle point of the primary remiges (Experiment 3). Experiment 6, which presents the wake behind the secondary remiges close to the root, is depicted in the Supplementary Material (Supplementary 2). In general, the common feature for all the wakes examined is that the concentrated regions of spanwise vorticity are small, suggesting that small scales dominate the wake flow. This qualitative examination of the reconstructed wakes shows a different topography of the vorticity field in comparison to other birds that have been tested in the same facility (Gurka et al. 2017): the near wake flow of passerine such as European starling (*S.vulgaris*) and America robin (*Turdus migratorius*) and a shorebird (western sandpiper, *C.mauri*) exhibit an organized wake where shedding is observed, although these birds are of different size and flight behavior. Furthermore, bird wakes studied in different facilities over the years have demonstrated organized wakes where shedding was observed independent of the flight mode or the bird species (see Supplementary 1). This discrepancy suggests that owls generate a wake substantially different when compared with these other birds.

#### Wake topography characterization

**Fig. 6:**
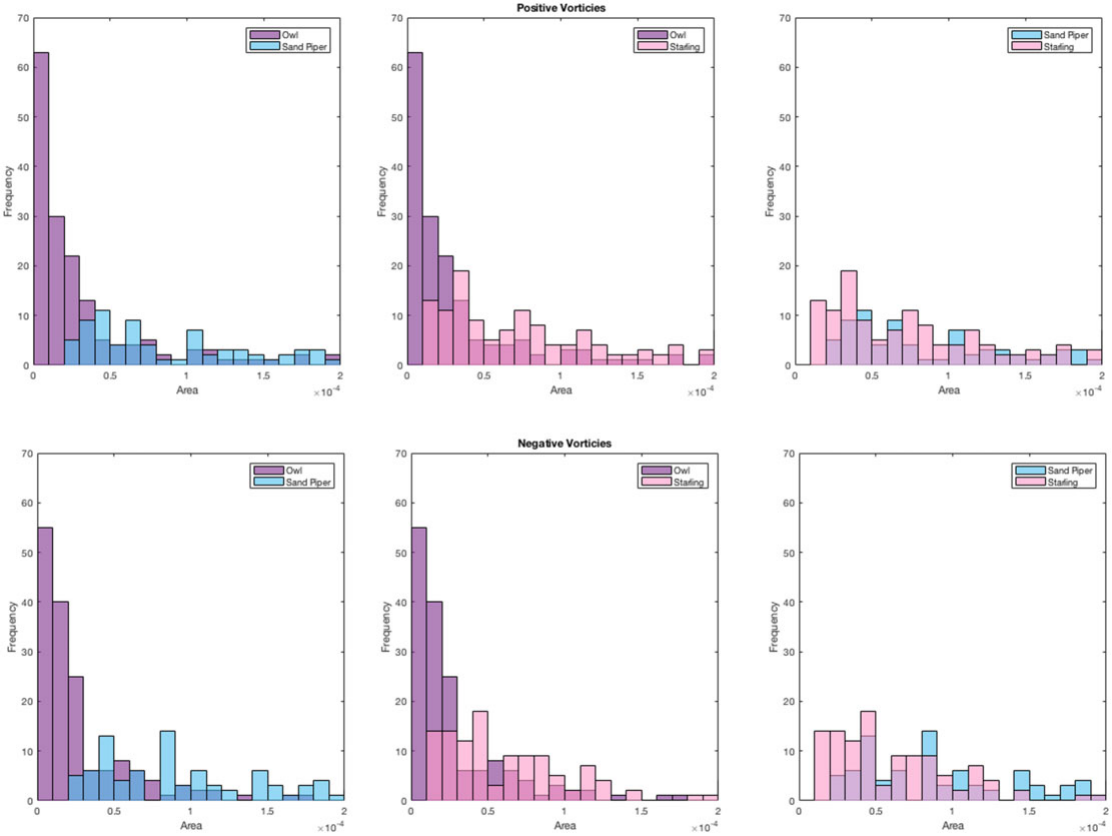
Histogram of the number of concentrated spanwise vorticity regions at the wake of the three birds. The histogram is based on blob analysis performed on the wake reconstruction contours appear in Fig. 5a for the owl and in Figs. 4 and 5 in Gurka et al. (2017) for the sandpiper and starling, respectively. The top figures illustrate the positive spanwise vorticity selections and the bottom showing the negative ones. The left figures compare the owl with the sandpiper, the middle compare it with the starling and the right figures compares the sandpiper with the starling.

In order to quantitatively characterize differences between the wakes of the boobook owl measured in our experiment and those typical of other birds, we performed a topographical and flow-scale analysis of the reconstructed wakes of the boobook owl, European starling, and western sandpiper as described in the “Materials and methods” section. We chose these two birds for comparison because they were flown at the same facility using the same measurement tools, and we have full access to their data. The results of the analysis of each species are compared and contrasted to enable a measure of the distinction of the wakes. Figure 6 depicts the histogram for Experiment 9 with the owl, along with the corresponding histograms for the starling and sandpiper wakes. The two subplots represent the histograms for the identified areas with positive vorticity (top figure) and the areas associated with negative vorticity (bottom figure). The histogram distribution for the starling and sandpiper appears to be similar, spanning a range of areas (0.2× 10^−4^ to 2.0×10^−^^4^ m^2^), with a large standard deviation. In comparison, the owl histogram is more narrowly distributed with a lower mean area than the other birds. These results are consistent over the range of experiments presented here (see Supplementary 2). The measured mean and standard deviation of the blob analysis histograms of the owl are smaller than the other two birds and are provided in Supplementary Table S1 in Supplementary 3. These results demonstrate that regions of large magnitude vorticity (*ω_z_c*/*U*_*∞*_ >|1|) in the wake of the owl tend to be smaller than those of other birds relative to their chord size. The limited large-scale motion in the owl’s wake suggests that large-scale motion may be suppressed/damped or not generated at all. This result is also consistent with the qualitative comparison of the wakes (Fig. 5a), where the wake reconstruction of the owl appears to exhibit a disorganized shedding compared with the starling and sandpiper.

#### Flow scale analysis

For the flow scale analysis, we estimated a characteristic flow scale using auto-correlation functions applied to the data in the near wake region (see details of the birds investigated in Table 3). The flow in the wake was unsteady and turbulent due to the intermediate Reynolds number. Smith et al. (1993) showed a linear relationship between concentrated regions of high vorticity and turbulent flow scales in homogenous turbulence (Smith et al. 1993). The characteristic turbulent scale is known as the integral lengthscale which is calculated from the auto-correlation function of the fluctuating velocity field with respect to a prescribed direction (Pope 2000). The wake developed behind the freely flying owl was unsteady and one cannot use the classical Reynolds decomposition in order to extract the velocity fluctuations. Therefore, we used Galilean decomposition of the velocity field as described in the “Materials and methods” section. Both longitudinal and transverse scales were calculated for the two velocity components and compared between the three birds, as shown in Table 4. The scales presented are averaged over each vector map and then over time: *L*_11_ is the longitudinal length scale for the streamwise velocity (*u*) in the streamwise direction (*x*) and *L*_22_ is the longitudinal length scale for the normal velocity (*v*) in the normal direction (*y*). The transverse scales (*L*_12_ and *L*_21_) correspond to the flow scales based on the streamwise velocity (*u*) and the normal velocity (*v*) along directions normal to each velocity component, respectively. Because the wake flow results from the wingbeat motion, the flow scales were estimated for two phases during the wingbeat cycle: the transition from DS to US (DSUS) and the transition from US to DS (USDS). The computed scales are normalized by the respective chord lengths (Table 4). The flow scales of the owl wake are smaller by an order of magnitude with respect to the wakes of the other two birds, whereas the flow scales of the wakes behind the starling and the sandpiper have similar magnitude. The flow scales do not seem to be dependent on the wingbeat phases. In addition, these flow scales are substantially smaller than the wing chord length and presumably are governed mainly by vorticity and/or strain. The smaller dominant flow scale found for the owl in comparison to the other birds is consistent with the results of the topographical analysis. The results of the flow scales and topographical analysis quantitatively demonstrate how the owl’s wake is fundamentally different from the two other birds.

**Table 3:** Morphological characteristics of the birds flown and the experimental parameters used for the fluid dynamic comparative analysis

	Mass (g)	Wingspan (cm)	Wing chord (cm)	Wingbeat frequency (Hz)	Wind speed (m/s)	Reynolds number	Strouhal number	Reduced frequency
Owl	273	90	13	6	8	66,200	0.35	0.31
Sandpiper	30.3	25.5	4.5	13	10	31,000	0.28	0.19
Starling	78	38.2	6	12.5	13.5	54,000	0.16	0.17

**Table 4:** Characteristic flow scales at the near wake based on the auto-correlation of the velocity fields

		Sandpiper		Starling		Owl	
Flow scale		DSUS^a^	USDS^b^	DSUS	USDS	DSUS	USDS
Longitudinal scale	*L*_11_ ^c^	0.19	0.24	0.16	0.15	0.06	0.07
	*L*_22_	0.26	0.18	0.13	0.18	0.07	0.09
Transverse scale	*L*_12_	0.14	0.16	0.14	0.11	0.06	0.08
	*L*_21_	0.18	0.23	0.13	0.11	0.06	0.09

*Note*: The scales are normalized by the respective wing chord length.

a DSUS, DS to US phase.

b USDS, US to DS phase.

c 1 and 2 refers to the streamwise and normal directions in the wind tunnel, respectively.

### Pressure distribution within the near wake

**Fig. 7:**
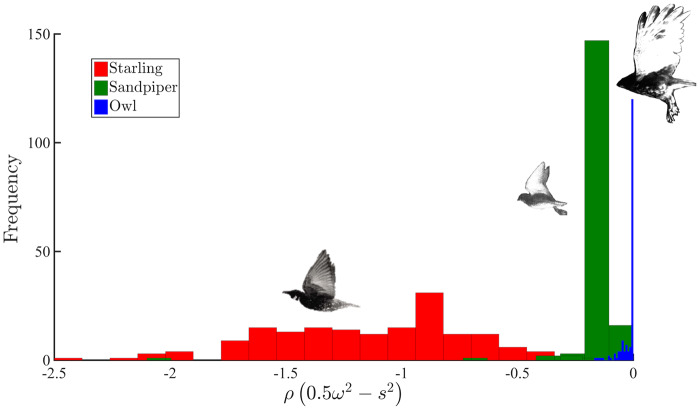
Histogram of the right-hand side term in Equation 1 (*x*-axis), which is the pressure Hessian (*ρ*[0.5*ω*^2^−*s*^2^]), where *ρ* is density, *ω* is vorticity, and *s* is strain-rate at the near wake region for the three birds. The histogram is based on calculating the vorticity and the strain fields for experiment #9 for the owl, and experimental data for the sandpiper and starling were deduced from Gurka et al. (2017). Blue, red, and green histograms correspond to the owl, starling, and sandpiper wake data, respectively.

To further explore the observed scale reduction, we examined the velocity gradient tensors; mainly the vorticity and strain in the wake region. We use the relation between pressure and velocity gradients through the pressure Hessian [see Equation (1)] that is derived from Navier–Stokes equations and play a dominant role in the vorticity equation, allowing to study the relation between pressure and velocity gradients, locally (Ohkitani and Kishiba 1995; Tsinober 2013). While this term provides an insight to the relation between vorticity and strain, it also provides an indirect estimate of the pressure field developed within the flow. Because aerodynamic noise is associated with pressure, it seems appropriate to examine the pressure Hessian, and compare it with that of other birds whose aeroacoustic signature is louder than that of an owl. Figure 7 presents a histogram distribution of the right-hand side of Equation (1) for the three birds, using the same data as the topographical and flow analyses. The right-hand side term of Equation (1) is calculated at the near wake region behind the wings, for all three birds in flapping mode about the wing mid-section. Therefore, it is plausible to assume that the locations of the measurements with respect to the wings in the spanwise and streamwise directions were similar and the phenomena observed occurred roughly at the same flow configuration. The blue colored histogram distribution in Fig. 7 corresponds to dataset obtained from Experiment 9 (owl 1), which has a mean value near zero with a tail ranging up to −0.25 kg/m^3^ s^2^. In contrast, for the starling, it ranges from 0 to −2.5 kg/m^3^ s^2^ (red histogram) and the sandpiper ranges from 0 to −0.5 kg/m^3^ s^2^ (green histogram); both with non-zero mean values. The histogram distribution for the starling is flatter, spans a wide range of values, and is similar to a normal distribution while the other two birds have a more skewed distribution, similar to log-normal distributions.

The difference between the distributions may be attributed to the wake flow patterns, which appear to be meandering for the owl and the sandpiper and less meandering for the starling, for the data cases studied herein. It is noteworthy that the same calculation was performed for the other owls’ datasets and all had similar distributions to the one presented in Fig. 7 (blue color histogram) with a similar range of values (see Supplementary Fig. S2 in Supplementary 2). Calculating 95% confidence intervals indicates that there is no overlap in the mean gradient pressure distributions among the owl (*m* ±SD =−0.018 ± 0.032, 95% CI −0.022 to −0.013), sandpiper (*m* ±SD =−0.14 ± 0.16, 95% CI −0.17 to −0.12), and the starling (*m* ±SD =−1.14 ± 0.43, 95% CI −1.21 to −1.08), where *m* is the mean, SD is the standard deviation, and CI is the confidence interval. These statistics show that the distribution of the pressure gradient of the owl’s wake is closer to zero (at least an order of magnitude smaller compared with the other birds) with little variation. This result indicates that the wake dynamics behind the owl are fundamentally different in comparison to the other two birds—consistent with the results of the other analyses. For the starling and sandpiper, the histogram mean values [right side of Equation (1)] are negative, on average. Here, *ω*^2^ corresponds to enstrophy and *s*^2^ is proportional to dissipation; thus, these results imply that dissipation is more dominant in the starling and sandpiper wakes behind the wing mid-span location, relative to the enstrophy [based on the relations between them in Equation (1)]. Yet, for the owl, the enstrophy is approximately double the dissipation as both terms counter each other to yield values in the wake that are close to zero. Therefore, we hypothesize that the owl, using its unique wing morphology, generates more vorticity than strain, which essentially is achieved by generating more small scales while destroying, or not generating, large scales in its wake.

## Discussion

The near wake flow dynamics of an owl feature unique characteristics that may be associated with its ability to fly silently. Our findings demonstrate significant differences between the wake of an owl and the wake of other birds (Spedding et al. 2003; Hedenström et al. 2006; Rosén et al. 2007; Henningsson et al. 2008; Johansson and Hedenström 2009; Tobalske et al. 2009; Altshuler et al. 2009; Hubel et al. 2010; Muijres et al. 2012; Kirchhefer et al. 2013; Gurka et al. 2017); these include bird wakes studied in various facilities over many years that have demonstrated organized wakes where shedding was observed independent of the flight mode or the bird species (see Supplementary 1).

The qualitative examination of the reconstructed wakes shows a different topography of the vorticity field in comparison to other birds that have been tested in the AFAR wind tunnel (Gurka et al. 2017): the near wake flow of passerines such as European starling (*S.vulgaris*) and America robin (*T.migratorius*), and a shorebird (western sandpiper, *C.mauri*) exhibit an organized wake where shedding is observed, although these birds are of different size and flight behavior. A detailed comparison between the owl wake and two birds: the western sandpiper and European starling were performed. While they are not comparable in size with the owl, they present broadly similar wake patterns although they differ in their flight behavior: long distance migratory, continuous flapping (sandpiper), and intermediate distance migratory, flap-gliding (starling).

In comparison to the other two birds, the wake of the owls is quantitatively different in terms of the scales of the flow. This result was confirmed by two different methods of estimation of the flow scales. The topographical analysis demonstrated that the owl’s wake is qualitatively more disorganized (no street is apparent) at the mid-span location of the wing and contain smaller areas of large magnitude of vorticity (|*ω_z_c*/*U_∞_*| > 1). This result is consistent with the notion that owls do not feature high aerodynamic performance (Kroeger et al. 1972; Geyer et al. 2017). An aerodynamic body is expected to generate an organized street in the wake (Gerrard 1966; Roshko 1993). Yet, such organized structures are absent in the owl’s wake. Furthermore, the flow scale analysis, which estimates the decorrelation scale of the flow patterns in the wake, indicates a smaller scale for the owl compared with the other two birds, consistent with the topographical analysis. The apparent absence of large flow scales may suggest that the turbulence production activity associated with these scales is somewhat limited (Pope 2000; Tsinober 2013). The dominance of small scales in the wake region also indicates an increase in the turbulence dissipation rate and vorticity. Together, this implies that over the wingbeat cycle, there is an imbalance between the production and dissipation of turbulence energy.

The aspects of the flow that result in the different distribution of scales were examined via the distribution of the two-dimensional pressure Hessian in the wake. The distribution for the owl shows that the pressure Hessian term has a mean near zero with a narrow distribution compared with the other two birds. The larger enstrophy in the owl’s wake relative to the dissipation (*s*^2^) further supports the results of smaller flow scales in the wake and/or suppression of larger scales. This result implies that in the owl’s wake the strain and vorticity fields interact with each other differently in comparison to the other two birds because the pressure Hessian describes the non-local interaction between vorticity and strain (Nomura and Post 1998). Tsinober (2013) suggested that when a flow field has a zero pressure Hessian, then the flow must be non-turbulent, or in other words, that nonlocality due to pressure is essential for (self-)sustaining turbulence. Therefore, the zero distribution suggests that turbulence is suppressed through distractive local interaction between vorticity and strain. The small mean pressure Hessian could be related to the noise suppression because noise and pressure are related, and we conjecture that the suppression of aerodynamic noise occurs through modulation of the flow scales in the wake.

Based on our findings we suggest that most of the owl’s wake has either (i) a strong three-dimensional motion in the spanwise direction (not measured) such that the wake behind the primary remiges is weakened relative to the tip region, or alternatively (ii) experienced a significant degradation of the turbulence level. For the former case it may be that these wake dynamics resulting from flow patterns formed above the wing section are shifted in the spanwise direction toward the wing tip such that the majority of momentum is transferred from the streamwise to the spanwise direction (as suggested originally by Kroeger et al. 1972). This shift would minimize the wake activity behind the majority of the wing by shifting all the momentum toward the tip region.

The degradation of turbulence may be the result of the unique morphological structure of the leading-edge serrations (Bachmann and Wagner 2011), velvety feathers surface, and trailing-edge fringes (Graham 1934) of the owl’s wing. Once the flow interacts with the wing at the leading edge, the serrations funnel the flow and presumably shift some of the momentum toward the tip; then the flow passes over the velvety feathers, forming pseudo-smooth surface; thus, maintaining lift and reducing friction (Bachmann et al. 2007). Subsequently, the flow passes toward the trailing edge. At the trailing edge, some of the fringes are oriented in the streamwise direction and some are oriented to overlap with the neighboring feathers (Bachmann et al. 2012), generate additional mixing due to their non-structured configuration. We suggest that this process causes the length scales of the flow to decrease dramatically, suppressing the large scales while producing more mixing and forming more small scales, which corresponds to additional generation of vorticity. This additional vorticity and/or suppression of larger flow length scales lead to a decreased pressure gradient field that can be associated with the aerodynamic noise. We suggest that the noise reduction is partially achieved by alternating the scales of the flow. Further research into how the various morphological features of the wings modify flow scales to balance the strain and vorticity fields in such a way that the pressure gradient field is minimized should be pursued.

## Ethics

The owls were brought from the “African Lion Safari” in Cambridge, Ontario, Canada under animal protocol number BOP-15-CS and protocol 2010-216 from the University of Western Ontario Animal Care committee.

## Data, code, and materials

All relevant data are given within the paper.

## Authors’ contributions

H.B.-G. and R.G. were responsible for the design of the experimental setup and performed the experiments and analyzed the data. R.G., J.L., and E.E.H. prepared the manuscript. J.L., H.B.-G., and K.K. performed the data analysis and edited the manuscript. G.A.K. assisted in the setup design and edited the manuscript. C.G. assisted with study design and data collection and edited the manuscript. G.M. was responsible for the capture, care, and training of the experimental birds.

## Supplementary Material

Supplement_Material_obz001
