# Supplementary material for: Flow Features of the Near Wake of the Australian Boobook Owl (Ninox boobook) During Flapping Flight Suggest an Aerodynamic Mechanism of Sound Suppression for Stealthy Flight
Source: Integr Org Biol. 2019 Feb 19;1(1):obz001. doi: 10.1093/iob/obz001 (PMC7671144; doi:10.1093/iob/obz001)
Supplement: Supplement_Material_obz001 [file supplement_material_obz001.zip › supplementary_1_owl_iob.pdf]

Supplementary 1: Vortex shedding at the wake of various birds' species extracted from PIV data in wind tunnel experiments

Bird	# specimen	# vector maps	# wingbeat	# recons.	Reynolds x 10^3	Speed [m/sec]	wingspan[m] /weight [kg]	identification
thrush nightingale[30]	4	8	1-2	2	13-35	4-11	0.13/0.030	$\omega_{spanwise}$
robin[31]	1	4	1 -2	2	13-29.8	4-10	0.11/0.016	$\omega_{spanwise}$
house martin[32]	1	6	1-2	2	25	4-10	0.15/0.017	$\omega_{spanwise}$
swift[33]	1	6	1	4	22	8.4	0.38/0.039	$\omega_{spanwise}$
blackcaps[34]	2	~100	2	1	17.5-29.5	6-10	0.42/0.016	$\omega_{streamwise}$
zebera Finch[35]	5	~90	1	1	35-85	6-10	N/A/0.017	$\omega_{spanwise,}$ $\omega_{streamwise}$
hummingbird[36]	1	8	1	3	N/A	2	N/A	ω_{normal}
lesser dog-faced fruit bat[37]	1	60	1.5	1	19-25	5-6.7	0.17/0.035	$swirl_{spanwise}$
pied flycatcher[38]	3	100	1	3	20	7	0.235/0.014	$\omega_{streamwise}$
starling[39]	2	45	1	5	49-60.6	12-13	0.38/0.078	$\omega_{spanwise}$
starling, sandpiper, robin[40]	3	150-200	2-3.5	3	54<br>31<br>86	13<br>10<br>10	0.38/0.078<br>0.26/0.030<br>0.43/0.078	$\omega_{spanwise}$
owl (current)	3	~100	2	9	66-71	8	0.7/0.270	$\omega_{spanwise}$
