# Supplementary material for: Flow Features of the Near Wake of the Australian Boobook Owl (Ninox boobook) During Flapping Flight Suggest an Aerodynamic Mechanism of Sound Suppression for Stealthy Flight
Source: Integr Org Biol. 2019 Feb 19;1(1):obz001. doi: 10.1093/iob/obz001 (PMC7671144; doi:10.1093/iob/obz001)
Supplement: Supplement_Material_obz001 [file supplement_material_obz001.zip › supplementary_2_owl_iob.pdf]

Supplementary 2: Reconstructed wakes for additional data sets and their pressure Hessian distribution

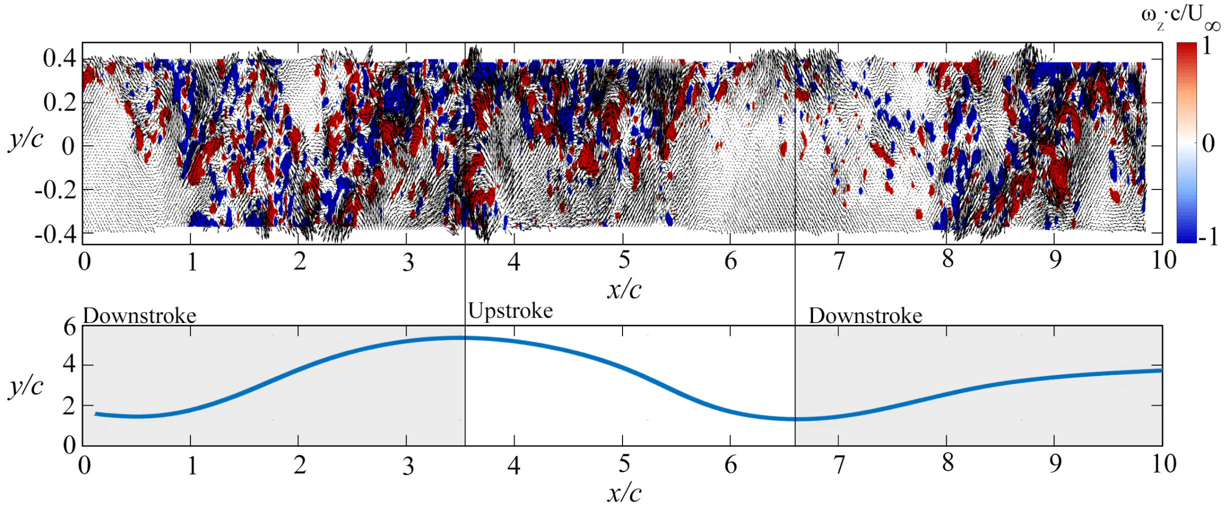

a) E1

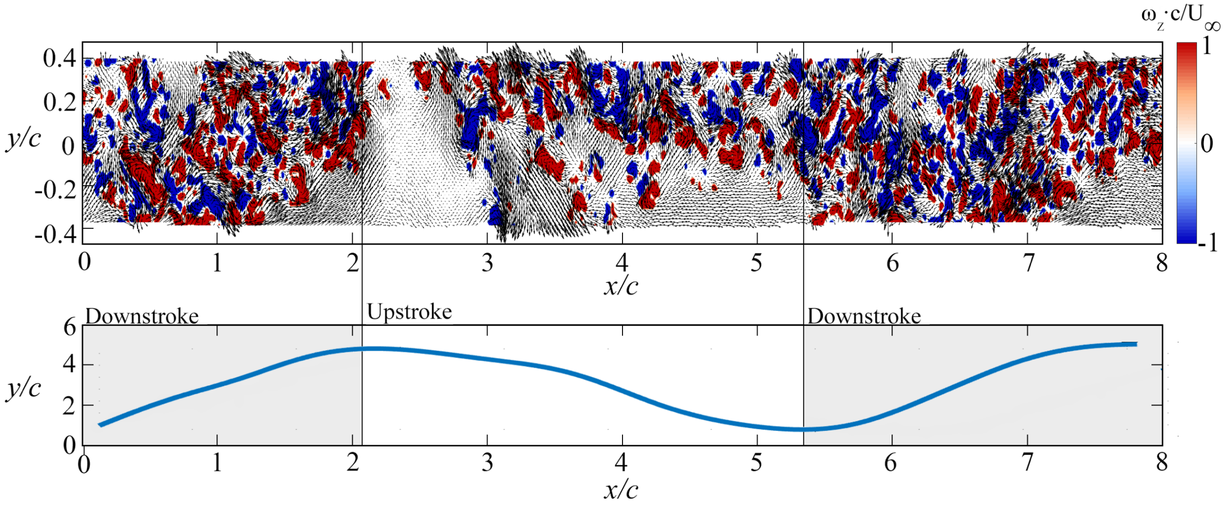

b) E2

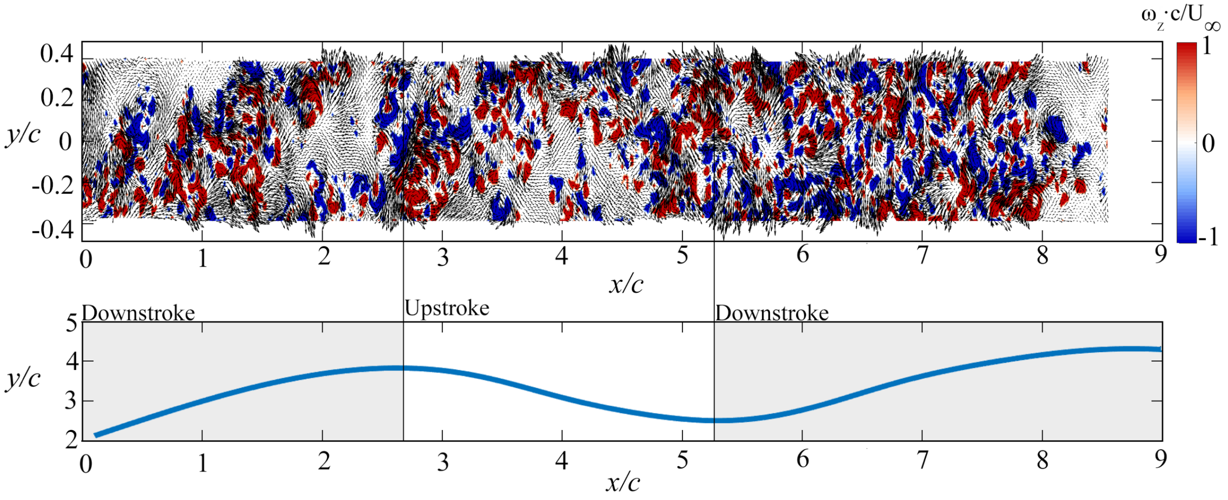

ω_z·c/U_∞
1
0
-1
0.4
0.2
y/c 0
-0.2
-0.4
0 1 2 3 4 5 6 7 8 9
x/c
Downstroke
Upstroke
Downstroke
5
4
y/c
3
2
0 1 2 3 4 5 6 7 8 9
x/c

c) E4

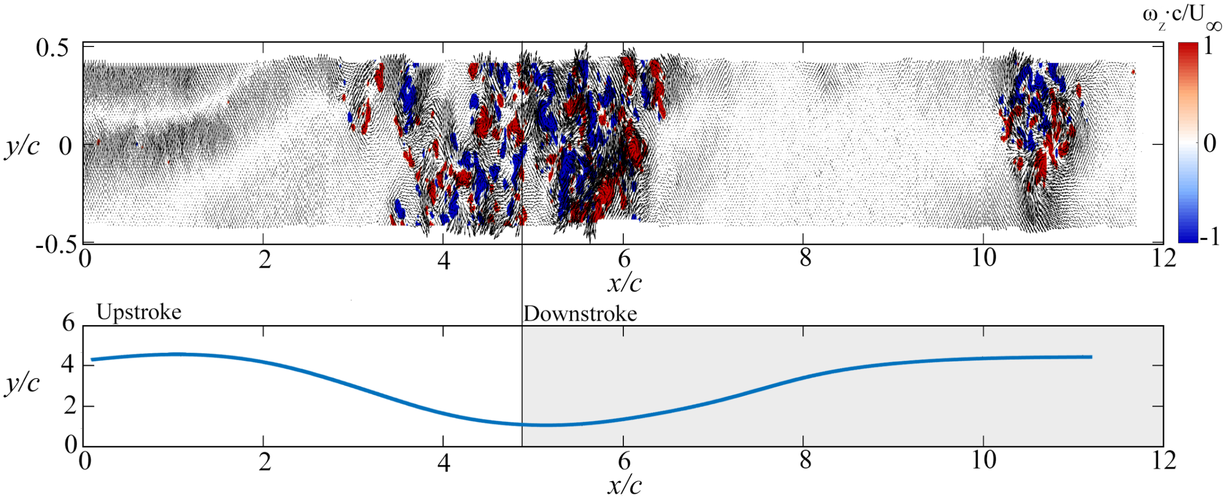

ω_z·c/U_∞
1
0
-1
0.5
y/c 0
-0.5
0 2 4 6 8 10 12
x/c
Upstroke
Downstroke
6
4
y/c
2
0
0 2 4 6 8 10 12
x/c

d) E5

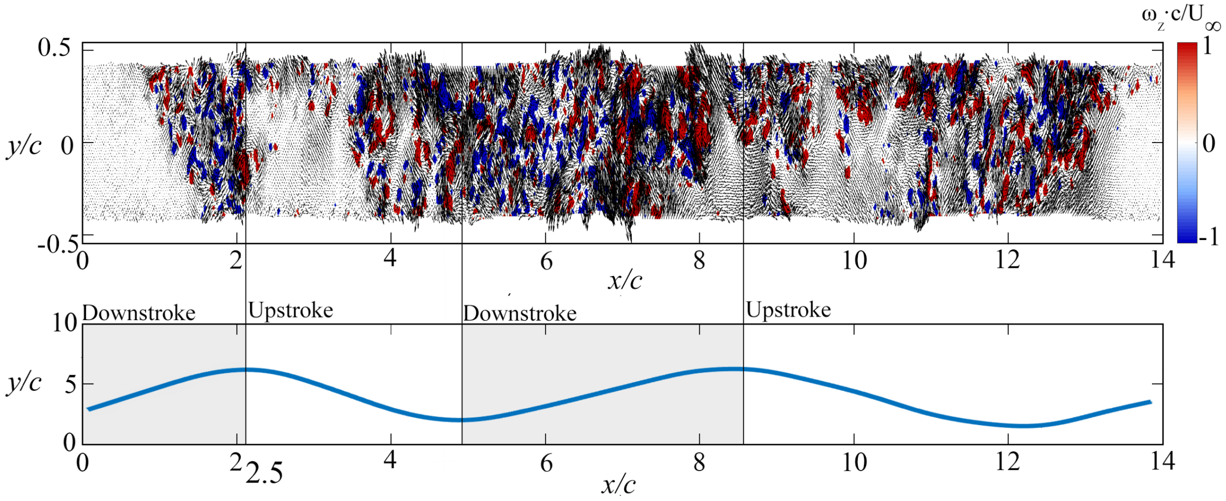

e) E7

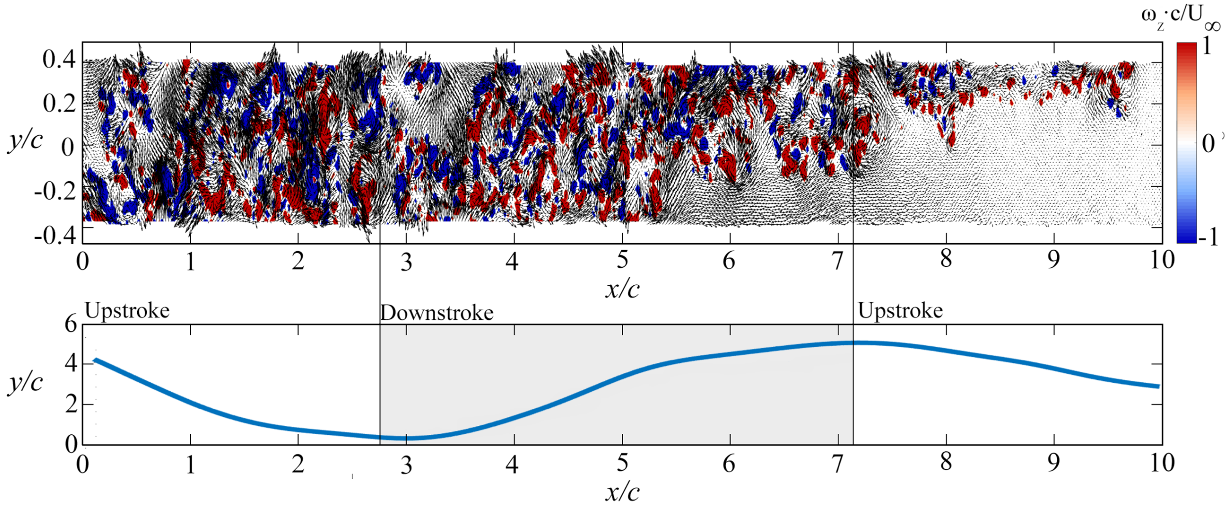

f) E6

Figure S1: Near-wake flow features of the boobook owl while flying in a flapping mode. The owl flew from right to left. (i) Wake reconstruction - The wake was sampled behind the wing at different spanwise sections: a,b,c) between the primary and secondary remiges: experiments #1, #2 and #4; d,e) outer region of the wing; the furthest location in the primary remiges: experiment #5 and #7; and f) secondary remiges close to the root: experiment #6. The contours represent the values of spanwise vorticity and the vectors depict the two-dimensional, two-component velocity field in the near wake. (ii) Wingtip displacement - The wingtip displacement is plotted against downstream chord length to directly correlate with the respective wake. The vertical black lines in

each graph represent the transition from upstroke to downstroke or downstroke to upstroke respectively.

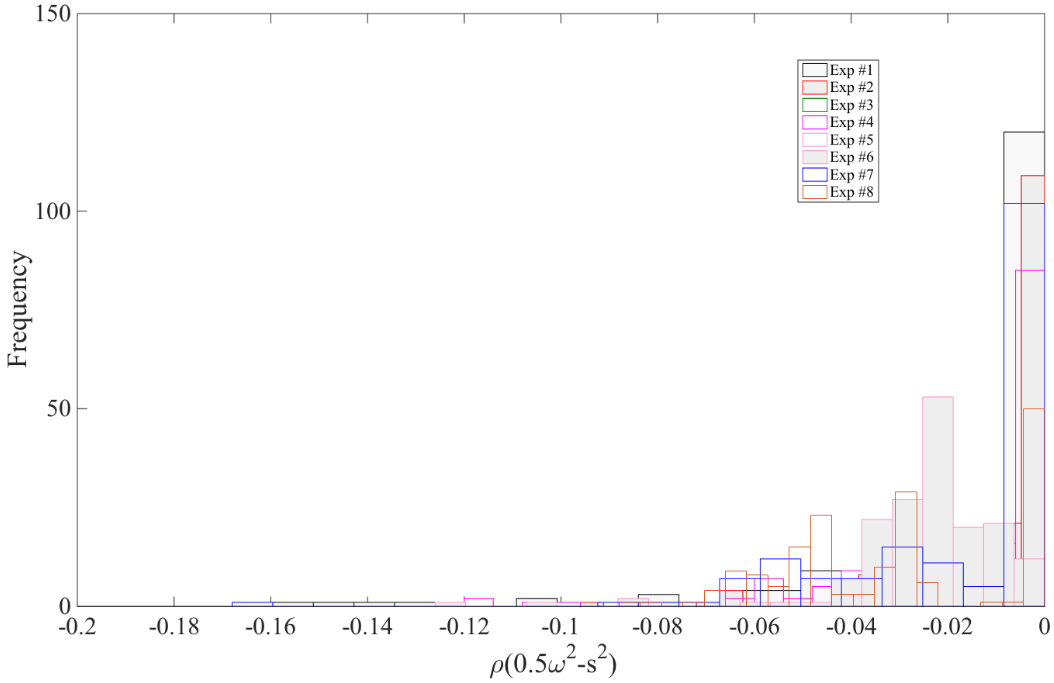

Figure S2: Distribution of the right-hand side term in equation 1; pressure Hessian at the near wake region for the owls. The histogram is based on calculating the vorticity and the strain fields for experiments #1-8.
