# Supplementary material for: Flow Features of the Near Wake of the Australian Boobook Owl (Ninox boobook) During Flapping Flight Suggest an Aerodynamic Mechanism of Sound Suppression for Stealthy Flight
Source: Integr Org Biol. 2019 Feb 19;1(1):obz001. doi: 10.1093/iob/obz001 (PMC7671144; doi:10.1093/iob/obz001)
Supplement: Supplement_Material_obz001 [file supplement_material_obz001.zip › supplementary_3_owl_iob.pdf]

Supplementary 3: Blob analysis procedure

The following steps have been performed in order to obtain a quantitative comparison between the vorticity fields at the wake of the three different birds. The steps are based on the so-called "Blob" analysis. The procedure has been performed in Matlab.

1. The input image are the images depicted based on figure 5 in the manuscript: Near wake reconstructions of the spanwise vorticity.

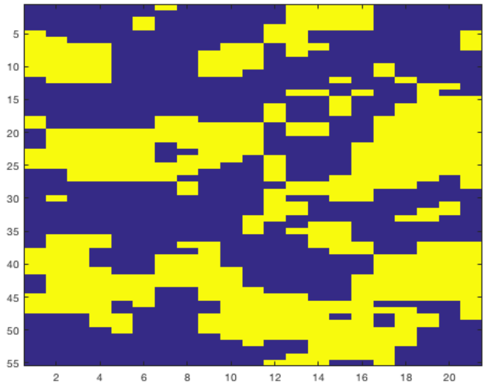

Figure S1: Portion of the vorticity field extracted from the near wake reconstruction of Experiment #9

2. The values of vorticity are separated into positive and negative ones. The image is than replotted using only positive values as well as another image is plotted using only negative ones. The data is already normalized, therefore, a threshold of values greater than 1 applied. Any value smaller than 1 is considered zero.

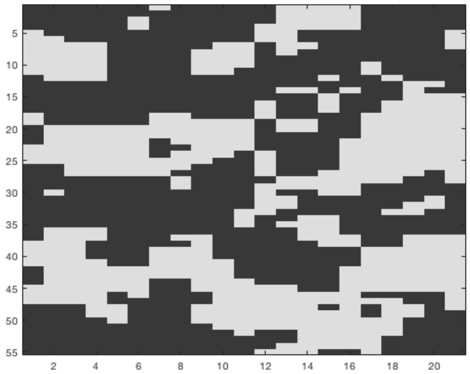

Figure S2: Converting the image vorticity contour to greyscale

3. The image is then converted into 8 bit grayscale image.

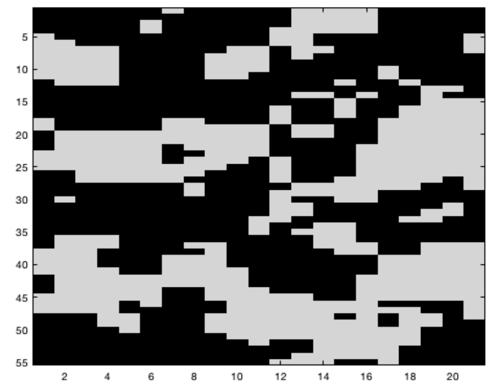

Figure S3: Adjustment of intensity values

4. Automatic image intensity adjustment is applied. This refer to converting the image values to fit the greyscale of 8 bit from 0-255.

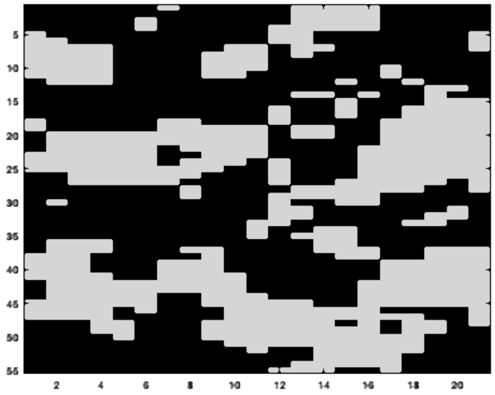

Figure S4: Image without background

5. Considering 0 to be the background. The background is identified and removed from the image.

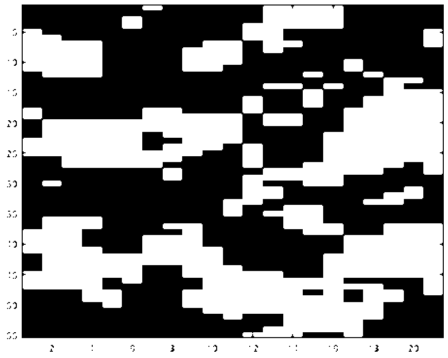

Figure S5: Image converted into a binary image

6. The image, once the background has been subtracted is converted into a binary one: values can obtain 0 or 1.

7. Border identification. It is assumed that a coherent vorticity structure (blob) is comprised of 8 pixels that are connected together (connectivity). Then, objects are identified such that if the connectivity is smaller than 8 it will not be considered, and if larger than 8, an object will be considered and the value (number of connected pixels) will be counted. Each object in the image will be flagged as a different color. Selecting the connectivity as smaller number (e.g., 4) yielded similar results but noisier. Filter is applied to remove objects that are identified that are not actually objects. The Filter removes objects that have eccentricity zero (essentially 1-pixel size) and additionally objects less than or equal to 3 pixels in area.

8. The script produces 2 figures, one before filtering and one after filtering with centroids plotted as black dots.

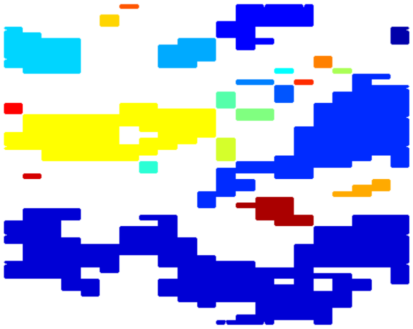

Figure S6: Border identification and objects selection

The parameters used to generate the data in the vorticity image as well as in the histogram were as follow:

Black and white images were created from the vorticity matrices

Thresholds for the images: i) of positive image > 1 = 1 and < 1 = 0 ii) of negative image < -1 = 1 and > -1 =0

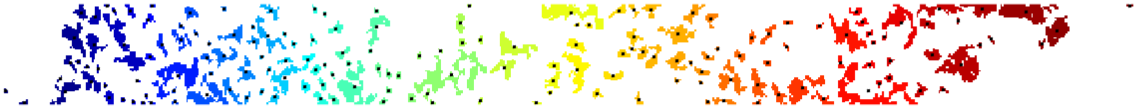

Figure S7: A color image with every object identified represented as a different color – and centroids of objects plotted as black dots, for example: (image is created from the positive values of Experiment #9)

Table S1: The measured mean and standard deviation of the blob analysis histograms of the owls, sandpiper and starling. The data corresponds to values depicted from the reconstructed vorticity field at the near wake and provide a measure of the vorticity structures size within the field. Wake data for Starling and Sandpiper is extracted from Gurka et al.[40]

Bird/Experiment	Positive vorticity		Negative vorticity	
	Mean	Standard deviation	Mean	Standard deviation
Owl				
Exp. #1	2.72×10^{-5}	4.22×10^{-5}	2.76×10^{-5}	5.20×10^{-5}
Exp. #2	3.22×10^{-5}	4.17×10^{-5}	2.68×10^{-5}	4.08×10^{-5}

Exp. #3	3.03×10^{-5}	5.22×10^{-5}	3.08×10^{-5}	5.83×10^{-5}
Exp. #4	2.58×10^{-5}	3.74×10^{-5}	2.25×10^{-5}	2.58×10^{-5}
Exp. #6	4.04×10^{-5}	6.29×10^{-5}	3.45×10^{-5}	6.38×10^{-5}
Exp. #7	3.59×10^{-5}	7.07×10^{-5}	3.16×10^{-5}	7.52×10^{-5}
Exp. #8	2.81×10^{-5}	2.88×10^{-5}	2.56×10^{-5}	2.99×10^{-5}
Exp. #9	3.60×10^{-5}	5.16×10^{-5}	3.70×10^{-5}	6.27×10^{-5}
Sandpiper				
Exp. #1	1.82×10^{-4}	2.69×10^{-4}	1.59×10^{-4}	2.79×10^{-4}
Exp. #2	1.39×10^{-4}	1.67×10^{-4}	1.65×10^{-4}	1.71×10^{-4}
Starling				
Exp. #1	1.72×10^{-4}	2.35×10^{-4}	2.09×10^{-4}	3.62×10^{-4}
Exp. #2	1.45×10^{-4}	1.86×10^{-4}	1.26×10^{-4}	1.86×10^{-4}
Exp. #3	1.61×10^{-4}	2.24×10^{-4}	1.89×10^{-4}	4.04×10^{-4}
